# Supplementary material for: Fructose reprogrammes glutamine-dependent oxidative metabolism to support LPS-induced inflammation
Source: Nat Commun. 2021 Feb 22;12:1209. doi: 10.1038/s41467-021-21461-4 (PMC7900179; doi:10.1038/s41467-021-21461-4)
Supplement: Supplementary file 1 — Supplementary Information [file 41467_2021_21461_MOESM1_ESM.pdf]

SUPPLEMENTARY INFORMATION

Title: Fructose reprograms glutamine-dependent oxidative metabolism to support LPS-induced inflammation

Authors: Nicholas Jones[1,7], Julianna Blagih[2,7], Fabio Zani[2], April Rees[1], David G. Hill[3], Benjamin J. Jenkins[1], Caroline J. Bull[3,4], Diana Moreira[5], Azari I. M. Bantan[1], James G. Cronin[1], Daniele Avancini[6], Gareth W. Jones[3], David K. Finlay[5], Karen H. Vousden[2], Emma E. Vincent[3,4,8*] and Catherine A. Thornton[1,8*]

[1]Institute of Life Science, Swansea University Medical School, Swansea University, Swansea, SA2 8PP, United Kingdom.

[2]The Francis Crick Institute, 1 Midland Road, London, NW1 1AT, United Kingdom.

[3]Cellular and Molecular Medicine, University of Bristol, Biomedical Sciences Building, Bristol, BS8 1TD, United Kingdom.

[4]MRC Integrative Epidemiology Unit, University of Bristol, Oakfield House, Bristol, BS8 2BN, United Kingdom.

[5]School of Biochemistry and Immunology, Trinity Biomedical Sciences Institute, Trinity College Dublin, 152-160 Pearce Street, Dublin, Ireland.

[6]San Raffaele Telethon Institute for Gene Therapy, San Raffaele Scientific Institute, Milan, 20132, Italy.

[7]These authors contributed equally

[8]These authors contributed equally

[*] Corresponding author: emma.vincent@bristol.ac.uk (E.E.V.)
c.a.thornton@swansea.ac.uk (C.A.T.)

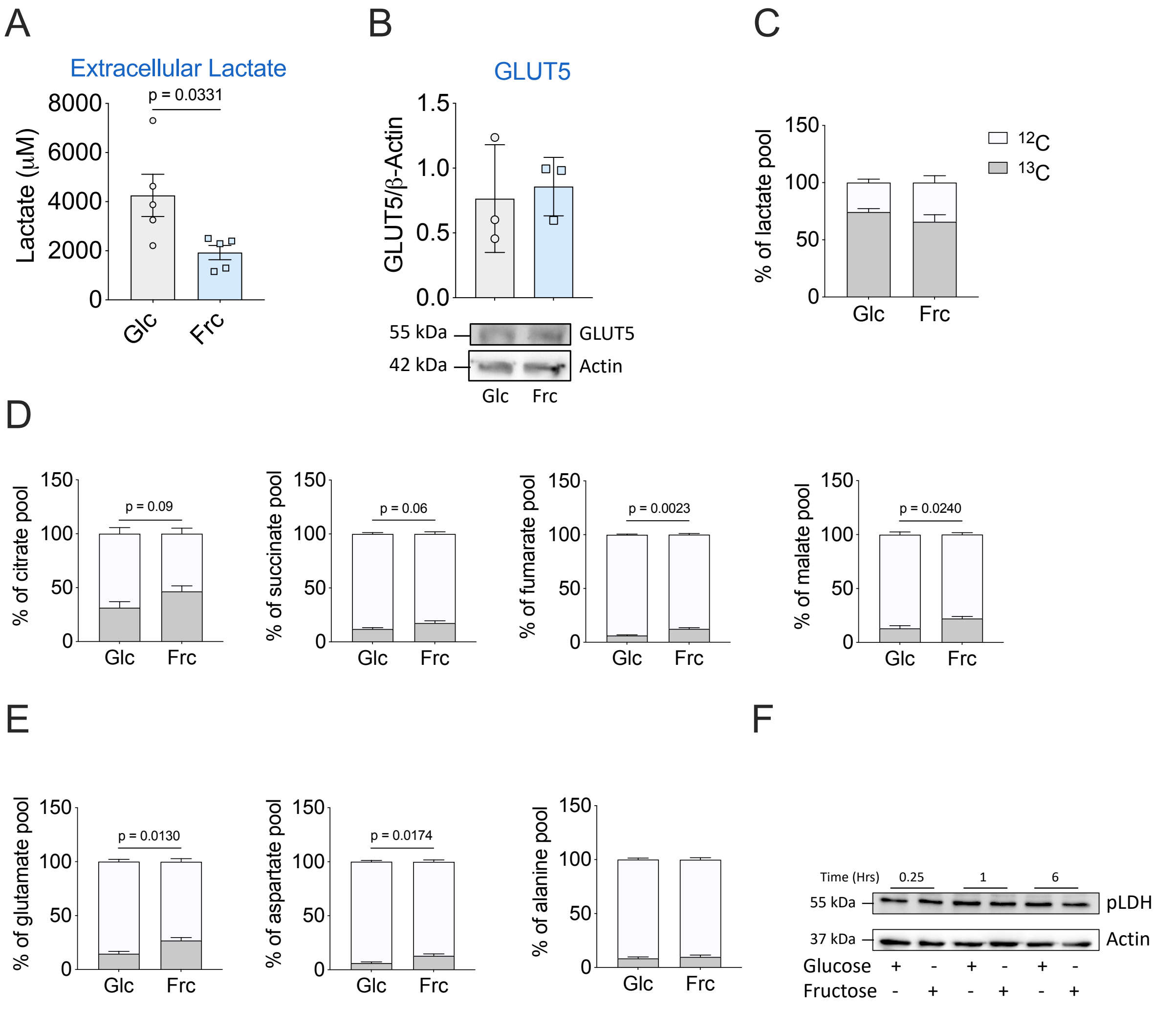

Supplementary Figure 1. Incorporation of fructose-derived carbons into lactate, TCA cycle and amino acid intermediates. (**a**) Extracellular levels of lactate produced in human monocytes (1.0 x10^6/mL) cultured with glucose or fructose for 24 hours in the presence of LPS (10 ng/mL). (**b**) Immunoblot of glucose transporter 5 (GLUT5) in monocytes cultured with glucose or fructose for 24 hours in the presence of LPS (10 ng/mL) with actin used as the housekeeping control. Percentage of pool of labelled $^{13}C_6$-glucose or $^{13}C_6$-fructose into (**c**) lactate, (**d**) TCA cycle intermediates, citrate, succinate, fumarate and malate or (**e**) amino acids glutamate, aspartate, alanine and proline. Statistical analysis performed on ^{13}C data only (**f**) Immunoblot of pLDH in monocytes cultured in glucose or fructose (11.1 mM) and LPS (10 ng/mL) for 0.25, 1 and 6 hours. Statistical significance was assessed using an unpaired, two-tailed t-test (**a-e**). Data representative of five (**a**), three (**b**), four (**c-e**) or two (**f**) independent experiments. Data are expressed as mean ± SEM. Source data are provided as a Source Datafile.

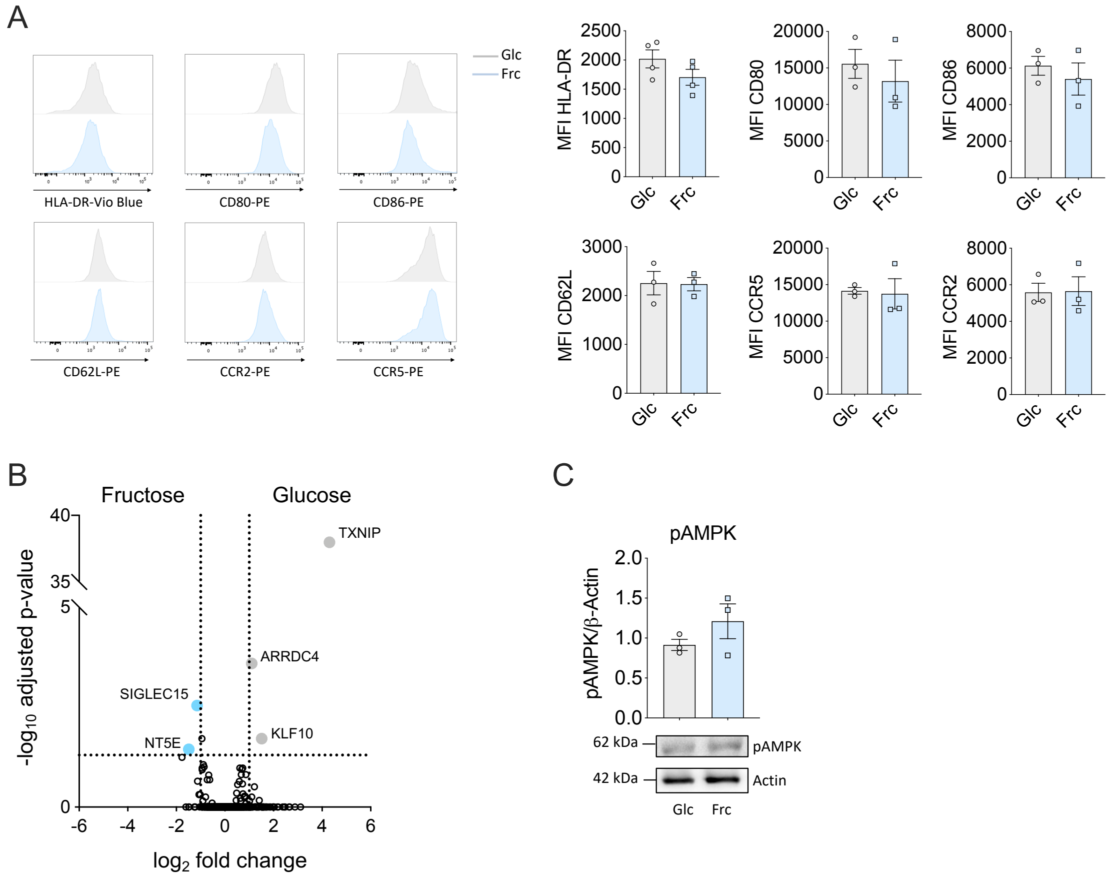

Supplementary Figure 2. Phenotypic surface markers and cytokine transcript levels are not affected by fructose culture. (**a**) Flow cytometry of phenotypic surface markers (HLA-DR, CD80, CD86, CD62L, CCR5 and CCR2) in monocytes cultured in glucose or fructose (11.1 mM) and LPS (10 ng/mL) for 24 hours. (**b**) Volcano plot showing the differentially regulated genes ($log_2FC>\pm1$, adjusted $P<0.05$; labelled) when comparing LPS-stimulated (10 ng/mL) monocytes treated with either glucose or fructose (11.1 mM). Genes significantly upregulated by glucose are coloured grey (n=3), and genes significantly upregulated by fructose are coloured blue (n=3). (**c**) Immunoblot of phospho-AMPK in monocytes cultured with glucose or fructose for 24 hours in the presence of LPS (10 ng/mL) with actin used as the housekeeping control. Statistical significance was assessed using an unpaired, two tailed t-test (**a, c**). Data are representative of three (**a**) with the exception of HLA-DR which is representative of four, or three (**b-c**) independent experiments and expressed as mean ± SEM. Source data are provided as a Source Datafile.

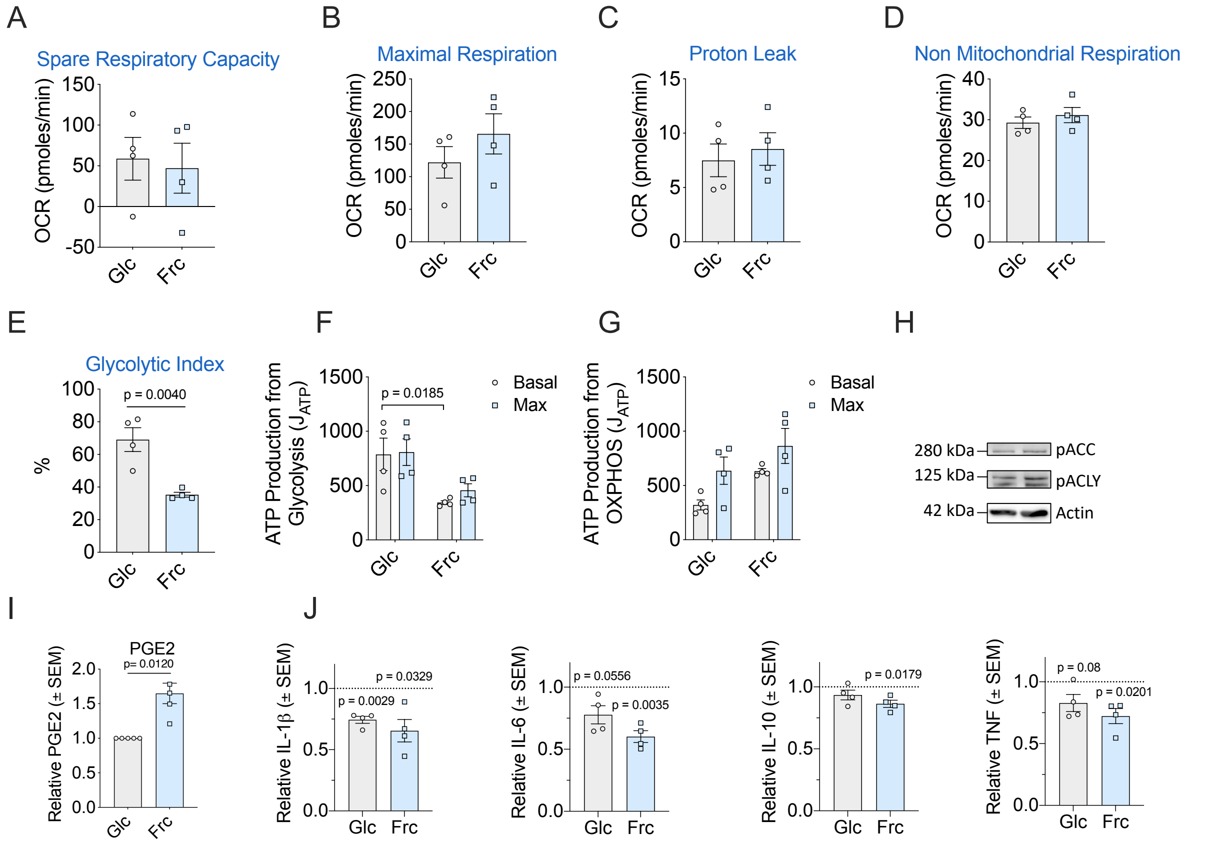

Supplementary Figure 3. Additional metabolic parameters. Oxidative parameters (**a**) spare respiratory capacity, (**b**) maximal respiration, (**c**) proton leak and (**d**) non mitochondrial respiration of monocytes cultured with glucose or fructose (11.1 mM) and stimulated with LPS (10 ng/mL) for 24 hours. (**e**) Percentage glycolytic index and ATP production rate (J_{ATP}) from (**f**) glycolysis or (**g**) oxidative phosphorylation. Monocytes cultured as above and (**h**) phosphorylation status of acetyl-CoA carboxylase (ACC) and ATP citrate lyase (ACLY) assessed by immunoblot with actin used as housekeeping control. (**i**) Extracellular release of prostaglandin E2 (PGE2) of monocytes cultured as previous. (**j**) Cytokine release (% of control) of IL-1β, IL-6, IL-10 and TNF (dotted line indicates control) of monocytes cultured in the presence or absence of ACLY inhibitor, BMS303141 (10 μM). Statistical significance was assessed using an unpaired, two-tailed t-test (**a-e, i**), two-way ANOVA with Sidak's multiple comparison test (**f-g**) or a one sample t test (**j**). Data representative of four (**a-g, j**), two (**h**) or five (**i**) independent experiments. Data are expressed as mean ± SEM. Source data are provided as a Source Datafile.

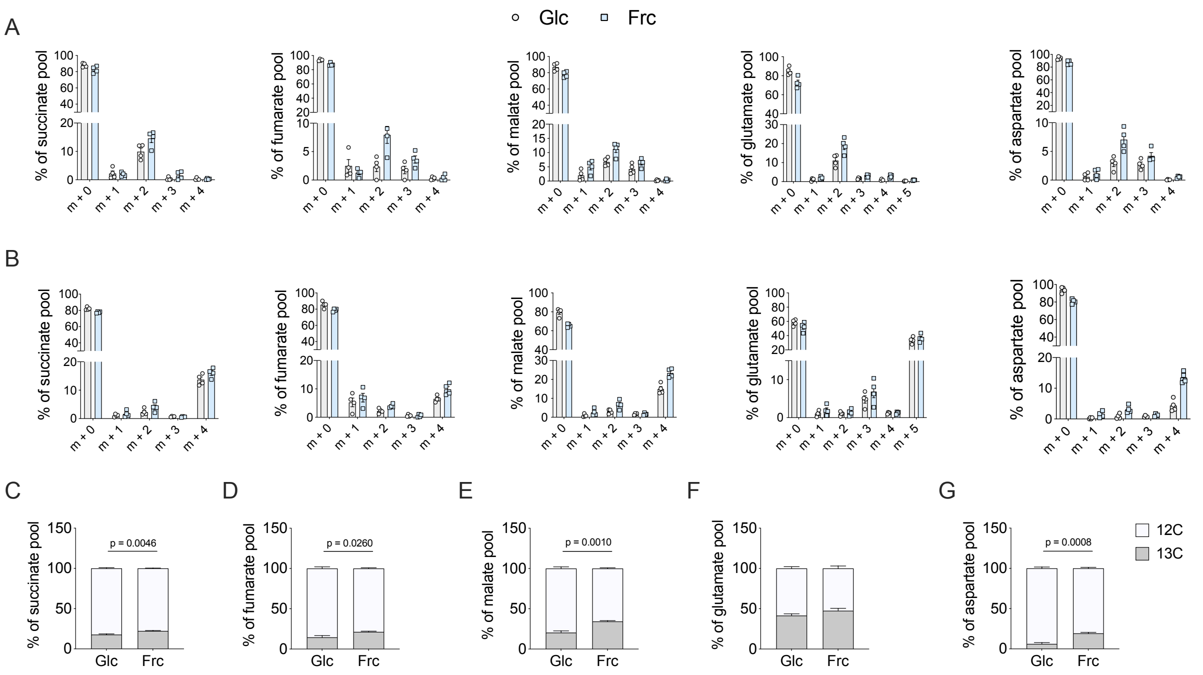

Supplementary Figure 4. Mass isotopologue distributions and incorporation of ^{13}C-glutamine into TCA cycle and amino acid metabolites. (**a**) Stable isotope tracing analysis (SITA) of 24-hour LPS-stimulated (10 ng/mL) monocytes cultured with uniformly labelled ^{13}C-glucose or ^{13}C-fructose into TCA cycle intermediates (succinate, fumarate and malate) and amino acids (glutamate and aspartate) with the mass isotopologue distribution (MID) represented as a % pool. (**b**) SITA of uniformly labelled ^{13}C-glutamine in the presence of ^{12}C-glucose or ^{12}C-fructose of 24-hour LPS-stimulated (10 ng/mL) monocytes with % MID shown. Stable isotope tracing of uniformly labelled ^{13}C-glutamine in the presence of ^{12}C-glucose or ^{12}C-fructose of 24-hour LPS-stimulated (10 ng/mL) monocytes. Percentage of pool of metabolites (**c**) succinate, (**d**) fumarate, (**e**) malate, (**f**) glutamate and (**g**) aspartate. Statistical significance for was assessed using an unpaired, two-tailed t test on the ^{13}C data (**c-g**). Data are representative of four (**a-c**) independent experiments. Data are expressed as mean ± SEM. Source data are provided as a Source Datafile.

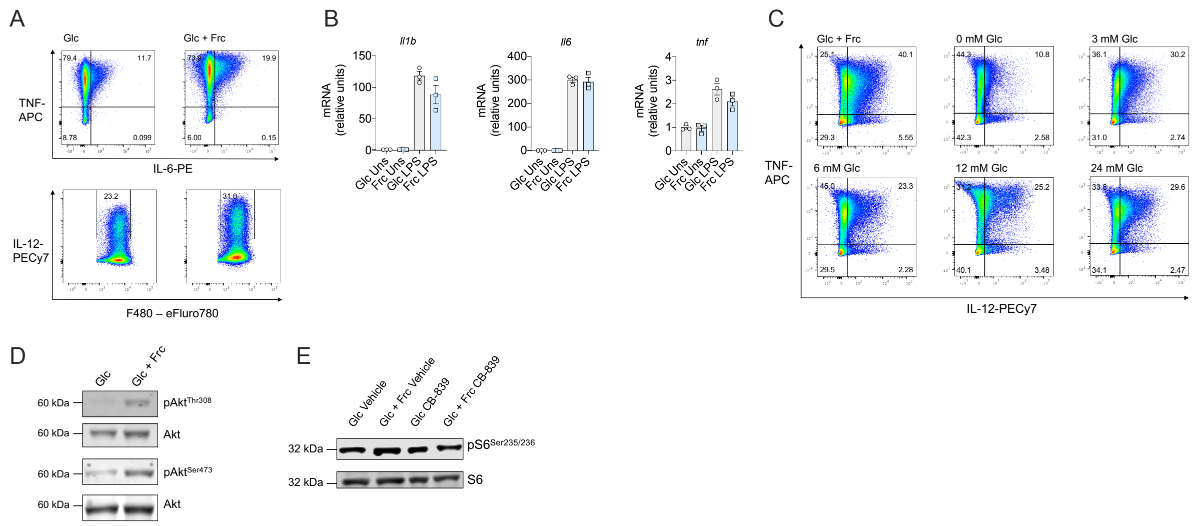

Supplementary Figure 5. Fructose in the presence of glucose enhances inflammation in macrophages (**a**) Flow cytometry analysis of LPS-stimulated (1 ng/ml) mouse macrophages cultured with glucose (24 mM) or glucose/fructose (both 12 mM) for intracellular cytokine production of IL-6 and IL-12. (**b**) qPCR for *Il1b, Il6 and Tnf* of mouse macrophages unstimulated or stimulated with LPS (1 ng/mL) for 18 hours cultured in either glucose (24 mM) or glucose/fructose (both 12 mM). (**c**) Intracellular cytokine staining (ICS) for TNF and IL12 in mouse macrophages cultured with varying glucose concentrations (24, 12, 6, 3 and 0 mM) stimulated with LPS (1 ng/ml) for 5 hours. (**d**) Immunoblot of phospho-Akt^{Thr308} and phospho-Akt^{Ser473} in macrophages cultured with glucose (24 mM) or glucose/fructose (both 12 mM) for 18 hours in the presence of LPS (1 ng/ml) with total Akt used as the housekeeping control. (**e**) Immunoblot analysis of p-$S6^{Ser235/236}$ in lysates from macrophages stimulated with 1 ng/ml of LPS and either cultured with glucose (24 mM) or glucose/fructose (both 12 mM) in the presence or absence of the glutaminase inhibitor, CB-839 (1 µM). Data are representative of four (**a, c**) or three (**b, d-e**) independent experiments. Data are expressed as mean ± SEM (**b**). Source data are provided as a Source Datafile.

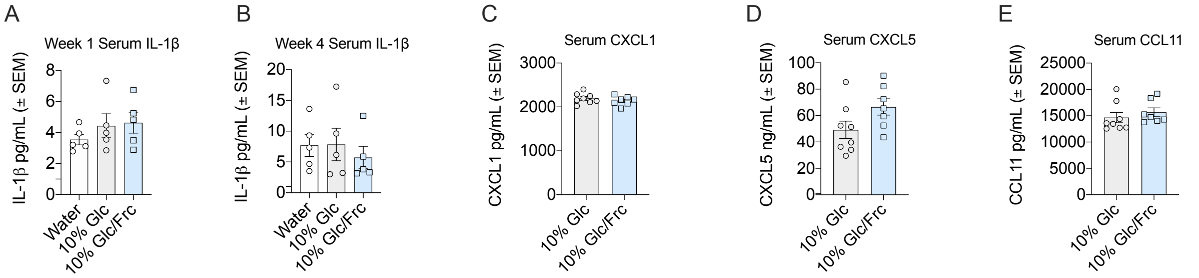

Supplementary Figure 6. Serum cytokine and chemokine production in unchallenged and LPS-challenged mice on sugar water. Serum IL-1β levels of control mice given *ad libitum* 10% glucose or 10% glucose-fructose water for (**a**) 1 week and (**b**) 4 weeks. Serum chemokines (**c**) CXCL1, (**d**) CXCL5 and (**e**) CCL11 production of mice provided 10% glucose or 10% glucose-fructose water for 2 weeks followed by intraperitoneal injection of 0.1 mg/kg LPS for 3 hours. Statistical significance was assessed using a one-way ANOVA with Tukey's multiple comparison test (**a-b**) or unpaired t-test (**c-e**). Data are representative of 5 (**a-b**) or 7-8 (**c-e**) independent experiments. Data are expressed as mean ± SEM. Source data are provided as a Source Datafile.

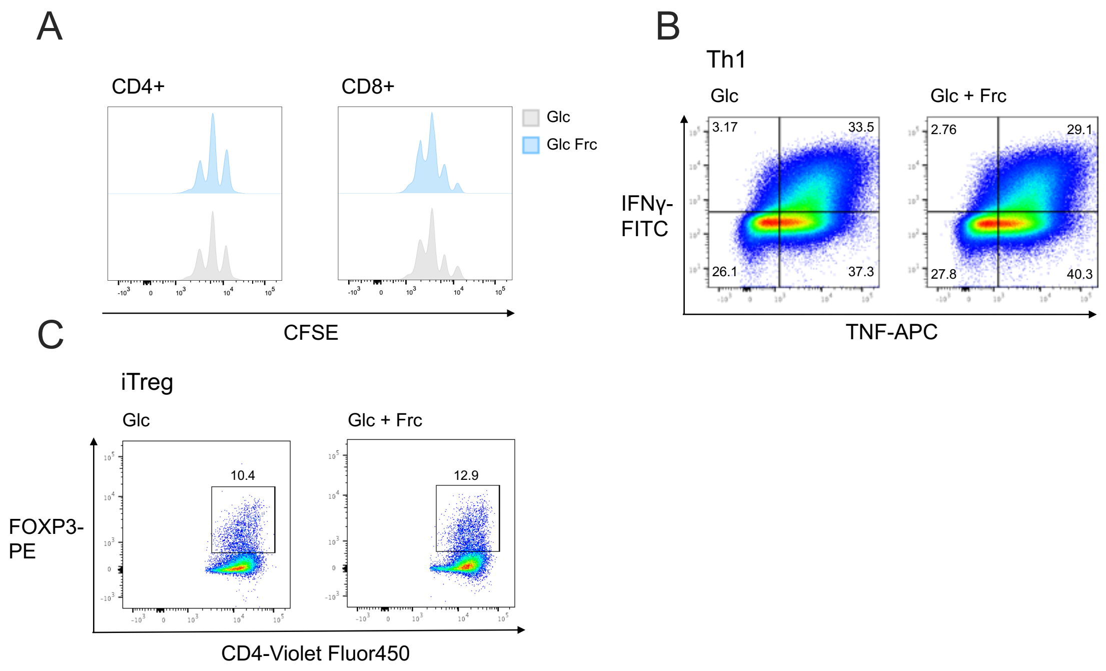

Supplementary Figure 7. Fructose in the presence of glucose does not affect T cell proliferation or CD4 T-cell polarization (**a**) Representative flow cytometry plots of CFSE-stained CD4+ or CD8+ T-cells cultured with glucose (24 mM) or glucose-fructose (both 12 mM) and activated with anti-CD3 (5 µg/ml) and anti-CD28 (2 µg/ml) for 72 hours. Dead cells were excluded using Fixable Viability dye eFluor780 (**b**) (**c**) Data are representative of 3 (**a-c**) independent experiments.

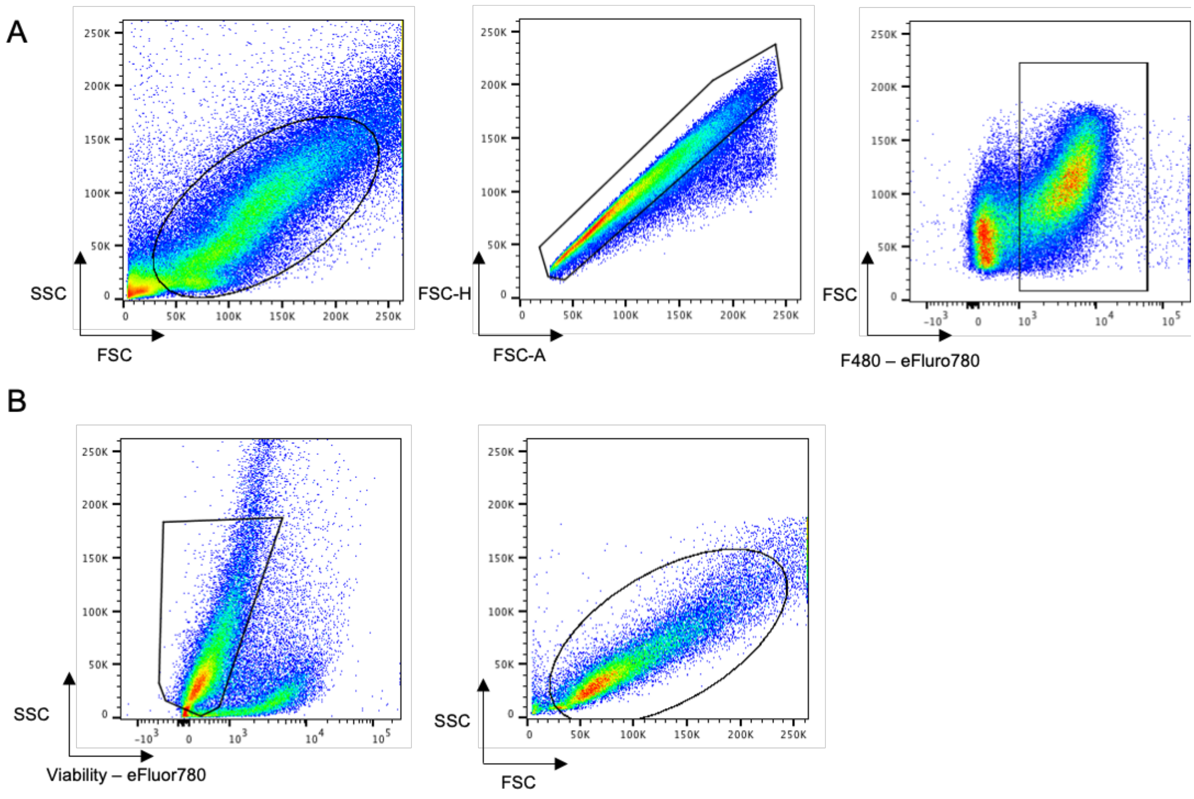

Supplementary Figure 8. Example gating strategies for macrophages. Gating strategy used to analyse mouse macrophages using **(a)** F480 eFluro780 (related to Figure 7A) or **(b)** dead cell exclusion using viability dye eFluor780 (related to Figure 7G).

Figure 3G

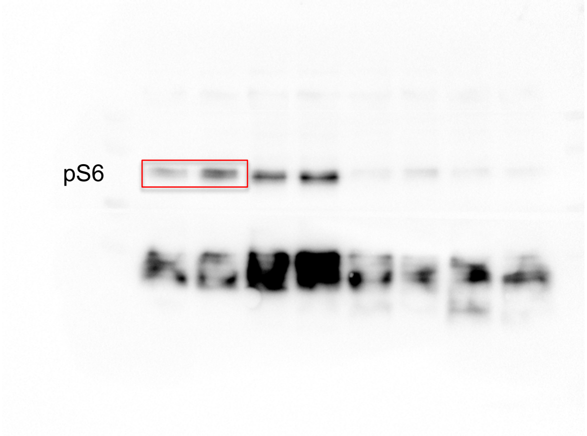

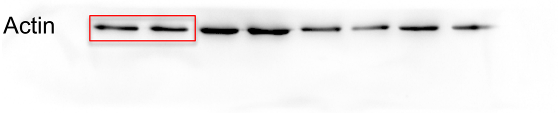

Figure 4J

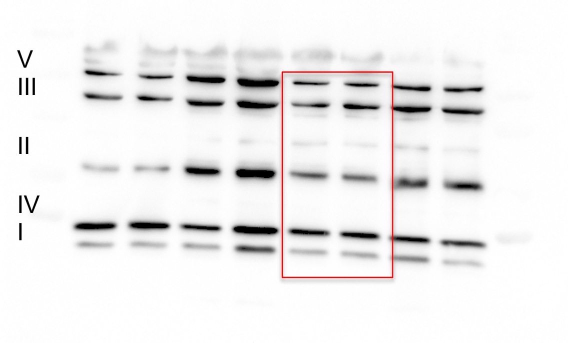

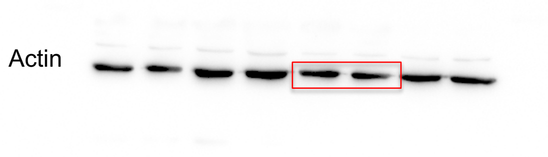

Figure 6A + B
Supplementary Figure 2C

HKI

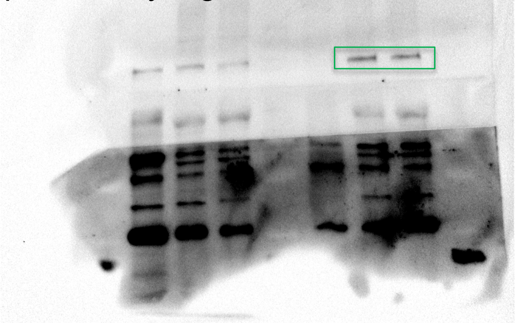

HKII

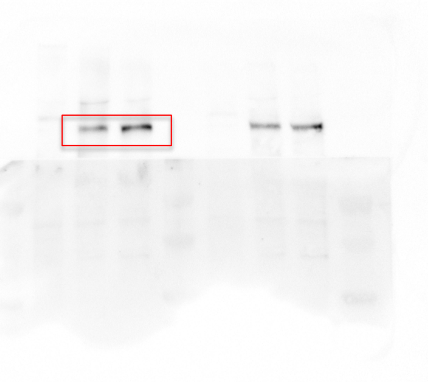

Actins - imaged together

Gel 1

Gel 2

Gel 3

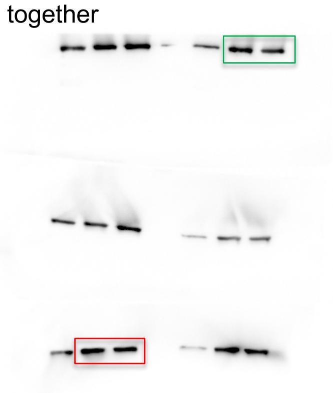

AMPK

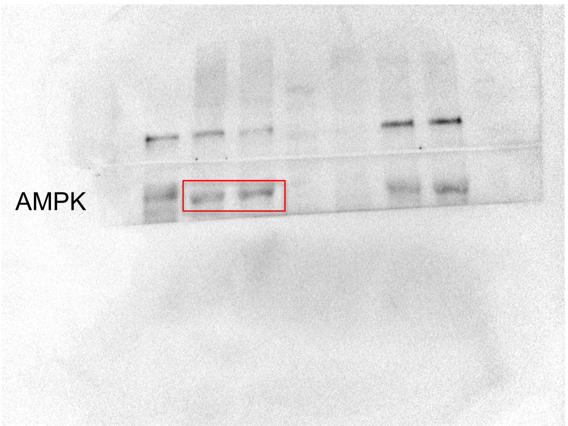

Figure 7E

pS6$^{Ser235/236}$

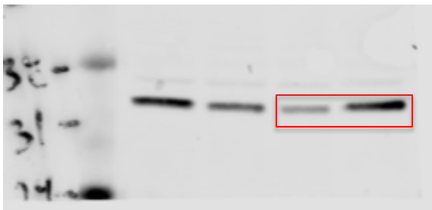

S6

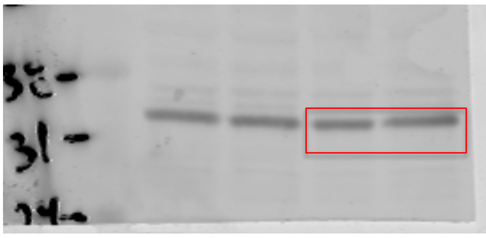

Supplementary Figure 1B

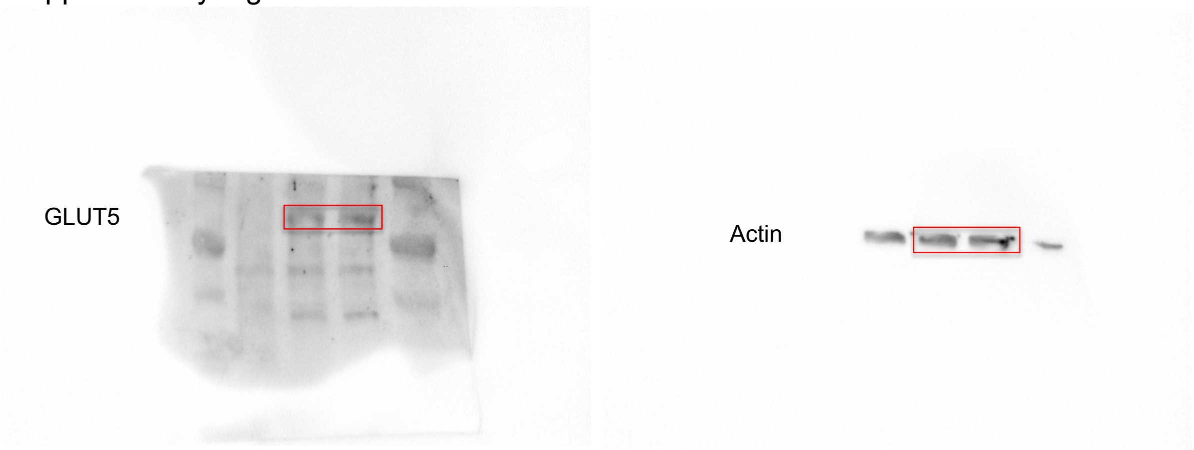

Supplementary Figure 1F

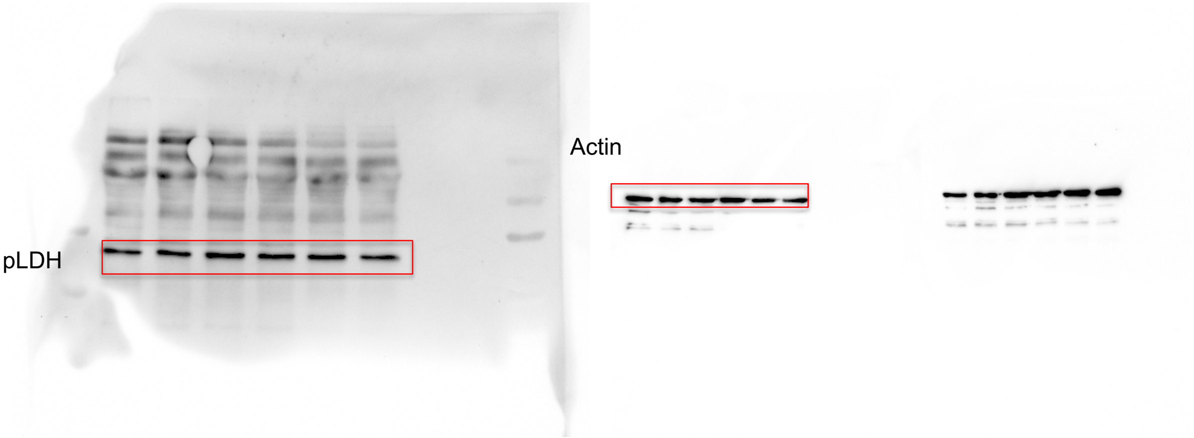

Supplementary Figure 3H

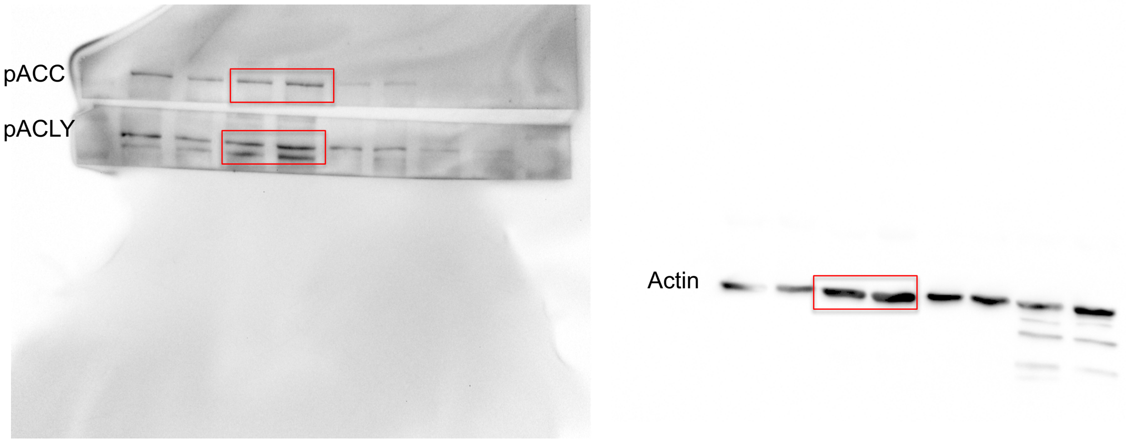

Supplementary Figure 5D

Akt^{Thr308}

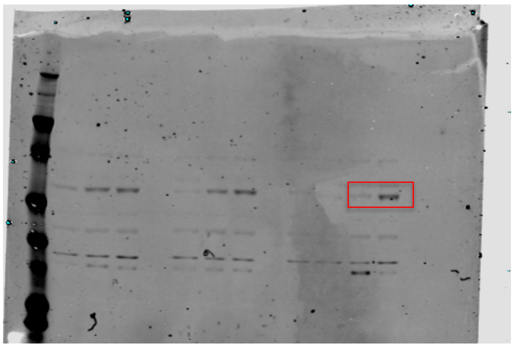

Akt^{Ser473}

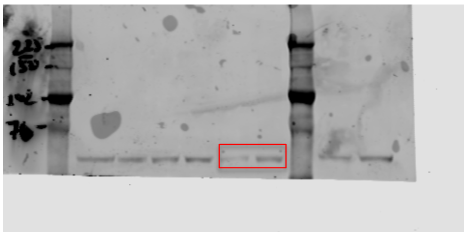

Akt

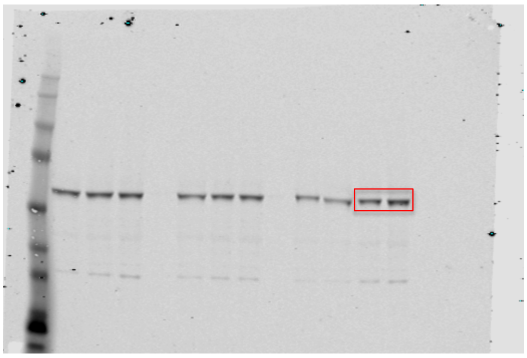

Akt

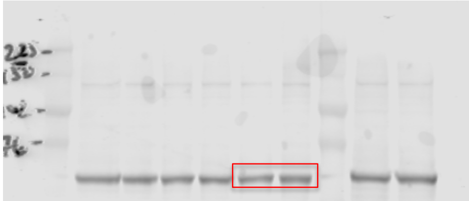

Supplementary Figure 5E

$pS6^{Ser235/236}$

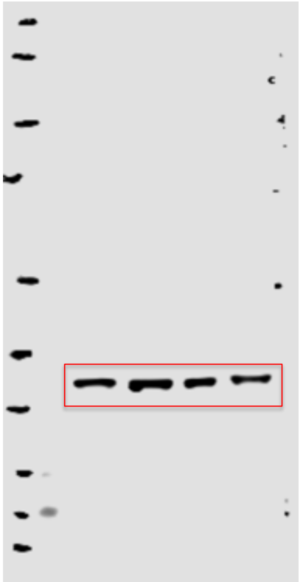

S6

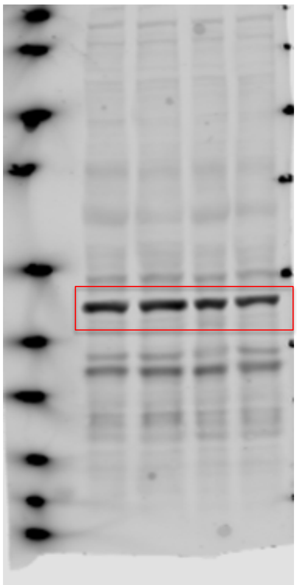

Supplementary Figure 9. Original uncropped immunoblots for main figures and supplementary figures.

Supplementary Table 1 - Primer sequences for qPCR

Gene	Forward primer	Reverse primer
Il6	TCCTACCCCAATTTCCAATGCTC	TTGGATGGTCTTGGTCCTTAGCC
Tnf	CCCCAAAGGGATGAGAAGTT	CTCCTCCACTTGGTGGTTTG
Il1b	GCAACTGTTCCTGAACTCAACT	TCTTTTGGGGTCCGTCAACT
cyc	ATGGTCAACCCCACCGTGT	TTTCTGCTGTCTTTGGAACTTTGTC
