# Supplementary material for: Fructose reprogrammes glutamine-dependent oxidative metabolism to support LPS-induced inflammation
Source: Nat Commun. 2021 Feb 22;12:1209. doi: 10.1038/s41467-021-21461-4 (PMC7900179; doi:10.1038/s41467-021-21461-4)
Supplement: Supplementary file 3 — Reporting Summary [file 41467_2021_21461_MOESM3_ESM.pdf]

nature research

Corresponding author(s): CA Thornton

Last updated by author(s): 30/12/20

Reporting Summary

Nature Research wishes to improve the reproducibility of the work that we publish. This form provides structure for consistency and transparency in reporting. For further information on Nature Research policies, see our Editorial Policies and the Editorial Policy Checklist.

Statistics

For all statistical analyses, confirm that the following items are present in the figure legend, table legend, main text, or Methods section.

n/a	Confirmed	
☐	☒	The exact sample size (*n*) for each experimental group/condition, given as a discrete number and unit of measurement
☐	☒	A statement on whether measurements were taken from distinct samples or whether the same sample was measured repeatedly
☐	☒	The statistical test(s) used AND whether they are one- or two-sided<br>*Only common tests should be described solely by name; describe more complex techniques in the Methods section.*
☒	☐	A description of all covariates tested
☐	☒	A description of any assumptions or corrections, such as tests of normality and adjustment for multiple comparisons
☐	☒	A full description of the statistical parameters including central tendency (e.g. means) or other basic estimates (e.g. regression coefficient) AND variation (e.g. standard deviation) or associated estimates of uncertainty (e.g. confidence intervals)
☐	☒	For null hypothesis testing, the test statistic (e.g. *F*, *t*, *r*) with confidence intervals, effect sizes, degrees of freedom and *P* value noted<br>*Give P values as exact values whenever suitable.*
☒	☐	For Bayesian analysis, information on the choice of priors and Markov chain Monte Carlo settings
☒	☐	For hierarchical and complex designs, identification of the appropriate level for tests and full reporting of outcomes
☒	☐	Estimates of effect sizes (e.g. Cohen's *d*, Pearson's *r*), indicating how they were calculated

Our web collection on statistics for biologists contains articles on many of the points above.

Software and code

Policy information about availability of computer code

Data collection	ImageLab (BioRad) V 5.2.1, NovoExpress V 1.4.1, Seahorse Wave Software V 2.6; Flow cytometry data was collected using the BD FACSDiva software V 8.0.1. Western blot fluorescence analysis was collected using the LiCor Image Studio Software V 5.2. Quantitative PCR data was collected using QuantStudio Design & Analysis software V 1.3.
Data analysis	GraphPad (Prism) V9, Mass spectrometry data: ChemStation E.02.02.1431, Immunoblot : Fiji ImageJ V 2.0.1, Flow cytometry data: FlowJo V 10, Thermo TraceFinder. RNA-seq analysis; Trimming – TrimGalore, Mapping – STAR (V 2.5.1b). Mapped to gene build GRCh38, QC – FastQC (V 0.11.2), Samtool (V 0.1.19) flagstat, Calculating counts – Subread featureCounts (V 1.5.1), Differential gene expression – Deseq2 (Bioconductor) and Heatmaps – Morpheus, https://software.broadinstitute.org/morpheus

For manuscripts utilizing custom algorithms or software that are central to the research but not yet described in published literature, software must be made available to editors and reviewers. We strongly encourage code deposition in a community repository (e.g. GitHub). See the Nature Research guidelines for submitting code & software for further information.

Data

Policy information about availability of data

All manuscripts must include a data availability statement. This statement should provide the following information, where applicable:

- Accession codes, unique identifiers, or web links for publicly available datasets
- A list of figures that have associated raw data
- A description of any restrictions on data availability

All data are available upon request and can be found within the manuscript and supplementary information. RNA-seq data have been deposited using the accession number GEO: GSE164058. Source data are provided with this manuscript.

Field-specific reporting

Please select the one below that is the best fit for your research. If you are not sure, read the appropriate sections before making your selection.

[x] Life sciences   [ ] Behavioural & social sciences   [ ] Ecological, evolutionary & environmental sciences

For a reference copy of the document with all sections, see nature.com/documents/nr-reporting-summary-flat.pdf

Life sciences study design

All studies must disclose on these points even when the disclosure is negative.

Sample size	No statistical test was used to determine sample size. For standard experimental procedures, sample sizes from our previous experience were used (Jones et al., 2019, Nat Comms). Experiments were performed using sample sizes based on standard protocols in the field (see Diehl et al. Nat Metab. 2019 Sep;1(9):861-867, Luengo et al. Nat Commun. 2019 Dec 6;10(1):5604).
Data exclusions	No data was excluded.
Replication	All experiments were replicated as described in the figure legend
Randomization	All animals used were aged 6-12 weeks and litter mates were randomly assigned to experimental groups. For human experiments no randomization was necessary as isolated monocytes were treated with either glucose or fructose.
Blinding	The investigator organizing the experimental groups and involved in sample collection was not blinded; however, colleagues aiding in data collection were blinded. For in vitro experiments, the investigators were not blinded for group allocation as the same investigator both planned and performed the experiment.

Reporting for specific materials, systems and methods

We require information from authors about some types of materials, experimental systems and methods used in many studies. Here, indicate whether each material, system or method listed is relevant to your study. If you are not sure if a list item applies to your research, read the appropriate section before selecting a response.

Materials & experimental systems

n/a	Involved in the study	
[ ]	[x]	Antibodies
[x]	[ ]	Eukaryotic cell lines
[x]	[ ]	Palaeontology and archaeology
[ ]	[x]	Animals and other organisms
[ ]	[x]	Human research participants
[x]	[ ]	Clinical data
[x]	[ ]	Dual use research of concern

Methods

n/a	Involved in the study	
[x]	[ ]	ChIP-seq
[ ]	[x]	Flow cytometry
[x]	[ ]	MRI-based neuroimaging

Antibodies

Antibodies used

Cell Signalling
hexokinase I (HKI; 2024)
hexokinase II (HKII; 2867)
phospho-S6 ribosomal protein (Ser235-236; 4858)
total S6 (2317)
pAktThr308 (4056)
pAktSer473 (4060)
Total Akt (2920)
pAMPK (2535)
pACLY (4331)
pACC (3661)
pLDH (8176)

Abcam
OXPHOS human cocktail (ab110411)
GLUT5 (ab41533)
β-actin (8226)

	LiCOR<br>IRDye®800CW Donkey anti-mouse (926-32212)<br>IRDye®680LT Donkey anti-Rabbi (926-68023)<br><br>Invitrogen<br>FITC IL-1b-pro (NJTEN3; 11-7114-82)<br><br>TONBO Bioscience<br>FITC-IFNg (clone XMG1.2; 35-7311)<br>PE-FOXP3 (clone 3G3; 50-5773)<br>Violet Fluor450-CD4 (clone RM4-5; 750042)<br><br>eBioscience Life Technologies<br>TNF-conjugated to APC (clone MP6-XT22; 506308)<br><br>BioLegend<br>anti-CD14 Pacific Blue (clone 63D3; 367122)<br>anti-CD62L PE (DREG-56; 304806)<br>PerCP-Cy5.5 F4/80 (clone BM8; 123128)<br>PeCy7 IL12/23 (clone C15.6; 505210)<br>PE IL-6 (MP5-20F3; 504504)<br><br>Miltenyi Biotec<br>anti-HLA-DR VioBlue (AC122; 130-095-293)<br>anti-CD80 PE (REA661; 130-110-270)<br>anti-CD86 PE (FM95; 130-113-572)<br>anti-CCR5 PE (REA245; 130-117-356)<br>anti-CCR2 PE (REA624; 130-109-595)
Validation	All antibodies are commercially available. Antibodies employed here in our manuscript were previously reported and routinely used for the application used. All companies used report quality control measures to ensure validity and reproducibility. Validation information and previous citations for each individual antibody are found in the data sheets provided by the company.

Animals and other organisms

Policy information about studies involving animals; ARRIVE guidelines recommended for reporting animal research

Laboratory animals	All laboratory animals used were of pure C57/N6J background, female-male mice ages 11 weeks. Mice used for the LPS-induced sepsis model were females aged 8 weeks. Mice allocated for bone marrow derived macrophages were mixed sex (male or female) with ages ranging from 8-14 weeks.
Wild animals	There were no wild animals used in this study.
Field-collected samples	No field-collected samples were used in this study.
Ethics oversight	Animal experiments were subject to ethical review by the Francis Crick Animal Welfare and Ethical Review Body and regulation by the UK Home Office project licence P319AE968. All mice were housed under conditions in line with the Home Office guidelines (UK).

Note that full information on the approval of the study protocol must also be provided in the manuscript.

Human research participants

Policy information about studies involving human research participants

Population characteristics	The study was performed on a population of healthy adults aged 18 - 70 years old and included men and women. Participants were excluded if they had an immune-mediated disease, cancer in the past 5 years or had current/recent symptoms of viral or other infection. Participants using medication, such as statins, with immune response modifying effects, were also excluded. All samples were collected between 0800 and 1200
Recruitment	Participants were recruited from the staff and student populations at Swansea University, Wales UK. Potential participants responded to ethics committee approved advertising by contacting the local clinical research facility. The clinical research facility oversaw recruitment through informed written consent in response to an ethically approved participant information sheet that explained the study. Participant recruitment was conducted by the Joint Clinical Research Facility at Swansea University with no selection bias.
Ethics oversight	This project was approved by Wales Research Ethics Committee 6 (approval 13/WA/0190) which is a committee within the Health Research Authority structure within the UK and equivalent to Institutional Review Board in USA

Note that full information on the approval of the study protocol must also be provided in the manuscript.

Flow Cytometry

Plots

Confirm that:

- [x] The axis labels state the marker and fluorochrome used (e.g. CD4-FITC).
- [x] The axis scales are clearly visible. Include numbers along axes only for bottom left plot of group (a 'group' is an analysis of identical markers).
- [x] All plots are contour plots with outliers or pseudocolor plots.
- [x] A numerical value for number of cells or percentage (with statistics) is provided.

Methodology

Sample preparation	Sample preparation of biological sources (human blood, murine bone marrow) is as described in the methods section of the manuscript
Instrument	Novocyte or BD Symphony
Software	NovoExpress v 1.4.1 or BD FACSDIVA V 8.0.1
Cell population abundance	Purity was in excess of 90% for each CD14+ monocyte population analysed post autoMACS separation
Gating strategy	Monocytes or BMDM were initially gated on FSC/SSC to exclude debris, then a gate identifying single cells was used (FSC-H v FSC-A). Monocytes or BMDM were then stained with a target of interest whereby gating was determined using an unstained v stained sample. Gating strategies are present in the supplementary information.

- [x] Tick this box to confirm that a figure exemplifying the gating strategy is provided in the Supplementary Information.
